# Gankyrin is a predictive and oncogenic factor in well-differentiated and dedifferentiated liposarcoma

**DOI:** 10.18632/oncotarget.2375

**Published:** 2014-08-21

**Authors:** Ju-Ae Hwang, Heung-Mo Yang, Doo-Pyo Hong, Sung-Yeon Joo, Yoon-La Choi, Joo-Hung Park, Alexander J. Lazar, Raphael E. Pollock, Dina Lev, Sung Joo Kim

**Affiliations:** ^1^ Transplantation Research Center, Samsung Biomedical Research Institute, Seoul, Republic of Korea; ^2^ Samsung Advanced Institute for Health Sciences & Technology, Graduate School, Department of Health Sciences & Technology, Sungkyunkwan University; ^3^ Department of Surgery, Samsung Medical Center, Samsung Biomedical Research Institute, Sungkyunkwan University School of Medicine, Seoul, Republic of Korea; ^4^ Department of Pathology, Samsung Medical Center, Samsung Biomedical Research Institute, Sungkyunkwan University School of Medicine, Seoul, Republic of Korea; ^5^ Department of Biology, Changwon National University, Changwon, Kyungnam, Republic of Korea; ^6^ Department of Cancer Biology, University of Texas MD Anderson Cancer Center, Houston, TX, USA; ^7^ Division of Surgical Oncology, James Comprehensive Cancer Center, Ohio State University, Columbus, OH, USA; ^8^ Sarcoma Research Center, Samsung Medical Center, Seoul, Republic of Korea

**Keywords:** Liposarcoma, 2-DE, Gankyrin, Predictive factor, Tumorigenesis

## Abstract

Liposarcoma is one of the most common histologic types of soft tissue sarcoma and is frequently an aggressive cancer with poor outcome. Hence, alternative approaches other than surgical excision are necessary to improve treatment of well-differentiated/dedifferentiated liposarcoma (WDLPS/DDLPS).

For this reason, we performed a two-dimensional gel electrophoresis (2-DE) and matrix-assisted laser desorption/ionization-time of flight mass spectrometry/mass spectrometry (MALDI-TOF/MS) analysis to identify new factors for WDLPS and DDLPS. Among the selected candidate proteins, gankyrin, known to be an oncoprotein, showed a significantly high level of expression pattern and inversely low expression of p53/p21 in WDLPS and DDLPS tissues, suggesting possible utility as a new predictive factor. Moreover, inhibition of gankyrin not only led to reduction of *in vitro* cell growth ability including cell proliferation, colony-formation, and migration, but also *in vivo* DDLPS cell tumorigenesis, perhaps via downregulation of the p53 tumor suppressor gene and its p21 target and also reduction of AKT/mTOR signal activation. This study identifies gankyrin, for the first time, as new potential predictive and oncogenic factor of WDLPS and DDLPS, suggesting the potential for service as a future LPS therapeutic approach.

## INTRODUCTION

Liposarcoma (LPS) is the most common soft tissue sarcoma subtype and accounts for at least 20 % of all sarcomas in adults [1]. LPS can be subtyped into the following three categories: atypical lipomatous tumor or well differentiated LPS (ATL-WDLPS LPS) / dedifferentiated LPS (DDLPS), myxoid/round cell LPS and pleomorphic LPS [2] based on clinicopathological and molecular genetic characteristics. Both WDLPS and DDLPS are characterized as containing ring-shaped or giant-rod chromosomes with amplified 12q13-q21 regions [3, 4]. The mouse double minute 2 (MDM2) gene is the most frequently amplified gene in WD/DDLPS (almost 100 % incidence). The cyclin-dependent kinase 4 (CDK4) gene is amplified in more than 90 % of cases. MDM2 and CDK4 are useful factors in the differential diagnosis of WDLPS and DDLPS [5, 6]. However, amplification and overexpression of MDM2 and CDK4 does not distinguish WDLPS from DDLPS [7]. Moreover, DDLPS is a biphasic neoplasm in which one component is WDLPS and the other component is a non-lipogenic sarcoma of varying histological grade. This histological subtype behaves more aggressively than WDLPS and has an estimated 5-year disease-specific survival of 44 %, which is less than the 93 % for WDLPS [8]. The local recurrence rate for retroperitoneal DDLPS tumors reaches 80~90 % in most series and distant metastatic relapse is observed in up to 30 % of cases [9, 10]. Therefore, although MDM2 and CDK4 appear to share a common genetic background, the etiologies of WDLPS and DDLPS are unclear and may be different.

Gankyrin (also called PSMD10) protein (hereafter gankyrin) consists of 7 ankyrin repeat domains and was initially identified as a component of the 26S proteasome [11]. Gankyrin can interact with various proteins, such as MDM2, CDK4, retinoblastoma protein (pRb), NF-kB, and RhoGDI [11-15]. Therefore, through these protein-protein interactions, gankyrin enhances cell proliferation, cell cycle progression and anti-apoptotic activity [16-18]. Gankyrin also appears to function as an oncoprotein at high protein levels [12, 13]. Gankyrin is expressed at high levels in hepatocellular carcinoma (HCC) compared to in normal hepatic tissue [19], which suggests that gankyrin may have an important role in the early stages of human hepatocarcinogenesis [20]. Although these reports suggest the importance of gankyrin in cell proliferation and tumorigenesis, the precise molecular function of gankyrin in liposarcomagenesis remains unknown.

Previous studies using gene expression profiling in liposarcoma have focused on comparing liposarcoma subtypes with normal adipose tissue [21, 22], usually without a comparative analysis of WDLPS and DDLPS protein levels in their respective progression and dedifferentiation signatures. Herein, we utilized a proteome analysis approach incorporating 2-dimensional electrophoresis (2-DE) to identify new factor for WDLPS and DDLPS. This approach demonstrated that gankyrin expression was observed in WDLPS and DDLPS and it was higher in DDLPS than in WDLPS tissues, suggesting that gankyrin may serve as a specific molecular signature for WDLPS and DDLPS. We also found that p53 and p21 were significantly downregulated in DDLPS tissues in concordance with gankyrin protein expression. Moreover, downstream signaling through Akt/mTOR activation was also significantly decreased by inhibition of gankyrin expression in DDLPS cell lines. Our data using *in vitro* proliferation, colony formation, and migration, as well as *in vivo* tumor cell proliferation and tumorigenesis assays, suggest the importance of gankyrin. These results support the possibility that gankyrin is a possible molecular therapeutic target for the treatment of WDLPS and DDLPS patients.

## RESULTS

### Comparison of well-differentiated liposarcoma (WDLPS) and dedifferentiated liposarcoma (DDLPS)

**Figure 1:**
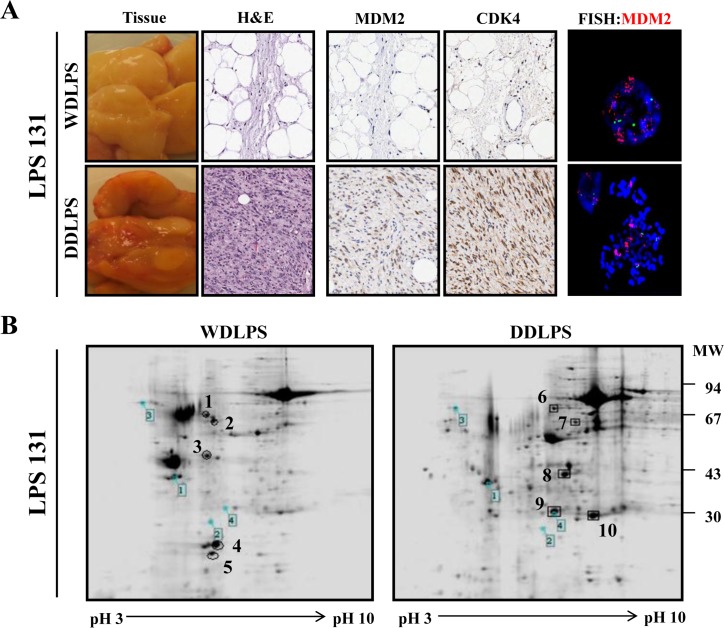
Characterization and 2-DE analysis of well-differentiated/dedifferentiated liposarcoma (WD/ DDLPS) (A) Representative images of WDLPS and DDLPS components by H&E staining, IHC staining for LPS markers (MDM2 and CDK4) and FISH analysis of MDM2 amplification in the WD/DDLPS specimens (LPS131). (B) Representative images of 2-DE gel with WDLPS and DDLPS components of WD/DDLPS specimens (LPS131). The gels were stained with coomassie brilliant blue R-250, and the boxed number is a landmark protein.

Fourteen LPS surgical specimens were prepared for proteome analysis. The clinical characteristics of the LPS patients are shown in supplemental table 1 (10 male and 4 female patients; average age of 54.2 years). The LPS subtypes in this proteome analysis consisted of WDLPS (n=4), DDLPS (n=4), and DDLPS containing WDLPS components (WD/DDLPS; n=6). The histological features of the LPS samples were confirmed with H&E staining, LPS marker expression (MDM2/CDK4), and MDM2-FISH analysis (Fig. 1A) prior to proteomic analysis. Two-dimensional electrophoresis (2-DE) analysis with WD/DDLPS samples (Fig. 1B) was used to compare protein expression levels in WDLPS and DDLPS. A total of 10 protein spots were selected from the 2-DE results based on the relative intergroup expression levels. There were 5 protein spots up-regulated in both WDLPS (spot numbers 1~5) and in DDLPS (spot numbers 6~10) (Fig. 1B). To identify the expressed proteins, the selected spots were analyzed by MALDI-TOF-MS. The spots were identified based on the sequence coverage, and each protein spot was given a suggested gene name (Table 1). The five protein spots that were increased in WDLPS were identified as vimentin, ATP synthase subunit beta (ATP5B), isoform 1 of heat shock cognate 71 kDa protein (HSPA8), isoform 1 of polymerase I and transcript release factor (PTRF), and annexin A2 isoform 1 (ANXA2). The five upregulated proteins in DDLPS were identified as Desmin, isoform gamma-B of fibrinogen gamma chain (FGG), myosin light chain 3 (MYL3), apolipoprotein A-I (APOA1) and PSMD10 26S proteasome non-ATPase regulatory subunit 10 (Gankyrin). These results suggest that LPS subtypes exhibit different patterns of protein expression at various differentiation stages. Therefore, different protein pools may play important and distinct roles in LPS biological functions.

**Table 1:** Identification of differentially expressed protein spots by MALDI-TOF-MS

	Protein No.	Accession No. (Swissprot)	Protein description	MW(kDa)	Sequence coverage^a^	Fold changes
Up-regulation in WDLPS	1	P08670	Vimentin	53.65	31.8	4.1
	2	P06576	ATP synthase subunit beta	24.42	56.9	4.6
	3	P11142-1	Isoform 1 of HS71	70.89	40.95	7.5
	4	Q6NZI2-1	PTRF Isoform 1 of Polymerase I	43.47	16.8	7.6
	5	P07355	annexin A2 isoform 1	38.6	65.3	3.0
Up-regulation in DDLPS	6	P17661	Desmin	53.3	30.9	3.0
	7	P02679-1	Isoform of Fibrinogen Gamma-B	51.51	30.7	2.0
	8	P08590	Myosin light chain 3	21.93	15.7	7.2
	9	P02647	Apolipoprotein A-I	30.77	67.1	7.2
	10	O75832	PSMD10 (Gankyrin)	24.42	25.9	8.9

### Dominent expression of gankyrin protein in dedifferentiated liposarcoma (DDLPS)

**Figure 2:**
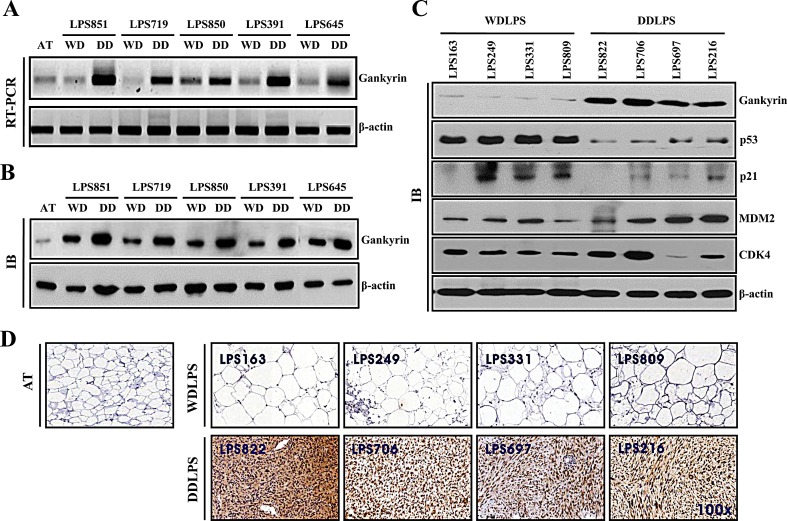
Comparison of protein expression levels in WDLPS and DDLPS Relative gankyrin mRNA levels (A) and protein levels (B) in each WDLPS (WD) and DDLPS (DD) component of WD/DDLPS specimens. AT is normal adipose tissue, and β-actin was used as an internal control. (C) Expression of proteins in WDLPS and DDLPS tissues were detected by immunoblot. (D) Immunohistochemical staining of gankyrin in pure WDLPS and DDLPS tissues. AT was used as a negative control (original magnification, 100x).

To determine the specific expression of candidate proteins in LPS tissues, we tested the expression levels of these proteins in the WDLPS and DDLPS components of WD/DDLPS specimens (n=5). We found that gankyrin was significantly upregulated in the DDLPS component of WD/DDLPS specimens at both the RNA level (Fig. 2A) and protein level (Fig. 2B). Furthermore, the upregulation of gankyrin was detected in WDLPS (n=4) and DDLPS (n=4) specimens (Fig. 2C). Gankyrin is known to regulate the degradation of p53 and to function as a proto-oncogene in hepatocellular carcinoma through interaction with MDM2. Therefore, the expression levels of MDM2, CDK4, p53 and p21 proteins were investigated to confirm whether gankyrin regulated p53 in WDLPS and DDLPS tissues. Although most LPS samples exhibited high levels of MDM2 and CDK4 expression, the variation of MDM2 and CDK4 protein levels in WDLPS and DDLPS were not significantly different. However, the protein level of p53 was significantly higher in WDLPS than in DDLPS and exhibited an inverse correlation with the expression of gankyrin in both WDLPS and DDLPS (Fig. 2C). The expression of p21, which is a direct downstream target of p53, was consistent with p53 expression (Fig. 2C). Gankyrin expression was then evaluated by immunohistochemistry in WDLPS and DDLPS specimens. As shown in figure 2D, gankyrin expression was positive in the nucleus and cytoplasm of DDLPS tissues but was negative in normal adipose tissue (AT) and WDLPS tissue. This result was also consistent with the gankyrin expression level determined by western blot analysis. These results suggest that gankyrin might be a dominant protein for DDLPS. Additionally, gankyrin expression may be related to p53 regulation in DDLPS.

### Association between gankyrin expression and patient prognosis

**Figure 3:**
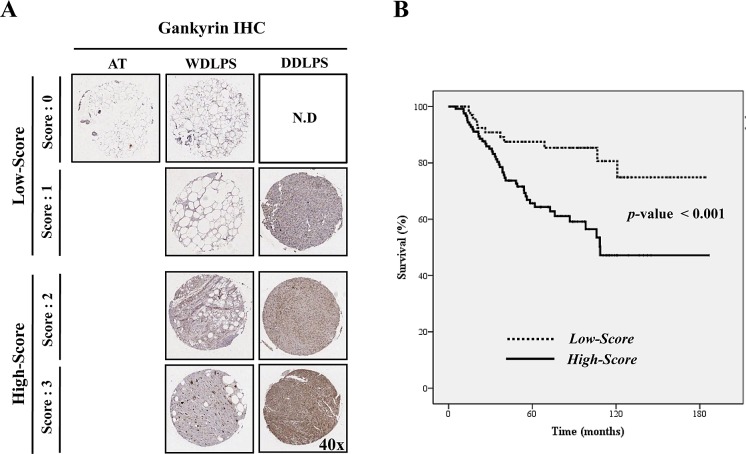
Relationship between gankyrin expression and LPS prognosis (A) Scoring of gankyrin in TMA of WD/DDLPS samples (original magnification, 40x). (B) The overall survival of 204 patients with WDLPS or DDLPS in gankyrin low-score (score 0-1) and high-score groups (score 2-3).N.D: not detected.

We next determined whether gankyrin expression was associated with WDLPS and DDLPS patient recurrence, metastasis and survival. Tissue microarrays (TMA) of WDLPS (n=123) and DDLPS (n=81) were prepared from 204 patient specimens and then examined by gankyrin immunostaining. Gankyrin expression levels were divided into four scoring grades (score 0 ~ 3), and the 204 LPS samples were divided into two groups: low-score (score 0-1) and high-score (score 2-3; Fig. 3A). Basal gankyrin expression was significantly higher in WDLPS and DDLPS (197 of 204 samples > score 0) than in normal adipose tissue. The proportion of high-score DDLPS (84.0 %) was also higher than that of WDLPS (50.4 %; P<0.001, Table 2). The high expression levels of gankyrin correlate with the likelihood of either recurrence (P=0.001) or metastasis (P=0.009, Table 2) of WDLPS and DDLPS. Moreover, although the prognostic significance of gankyrin is not easily derived in the DDLPS samples when overall survival of WDLPS and DDLPS patients were analyzed separately (Supplemental Fig. 2), Kaplan-Meier analysis revealed that WDLPS and DDLPS patients in the gankyrin high-score group exhibited significantly shorter survival rates than those of patients in the low-score group (*p* < 0.001, Fig. 3B). These results suggest that gankyrin expression is useful for determining the clinicopathological characteristics of WDLPS and DDLPS.

**Table 2:** Clinicopathological characters of WDLPS and DDLPS along with gankyrin expression

	IHC score of Gankyrin		*p*-value
	Low (N= 74)	High (N=130)	
**Histologic subtype**			
WDLPS	61 (49.6)	62 (50.4)	< 0.001
DDLPS	13 (16.0)	68 (84.0)	
**Tumor location**			
Trunk	48 (31.2)	106 (68.8)	0.008
Extremity	26 (52.0)	24 (48.0)	
**Tumor number**			
Unifocal	60 (40.3)	89 (59.7)	0.051
Multifocal	14 (25.5)	41 (74.5)	
**Recurrence**			
Yes	26 (25.2)	77 (74.8)	0.001
No	48 (48.5)	51 (51.5)	
**Metastasis**			
Yes	3 (12.5)	21 (87.5)	0.009
No	71 (39.9)	107 (60.1)	

Low: gankyrin score: 0~1, High: gankyrin score: 2~3

### Effect of gankyrin inhibition on the proliferation of LPS cell lines

**Figure 4:**
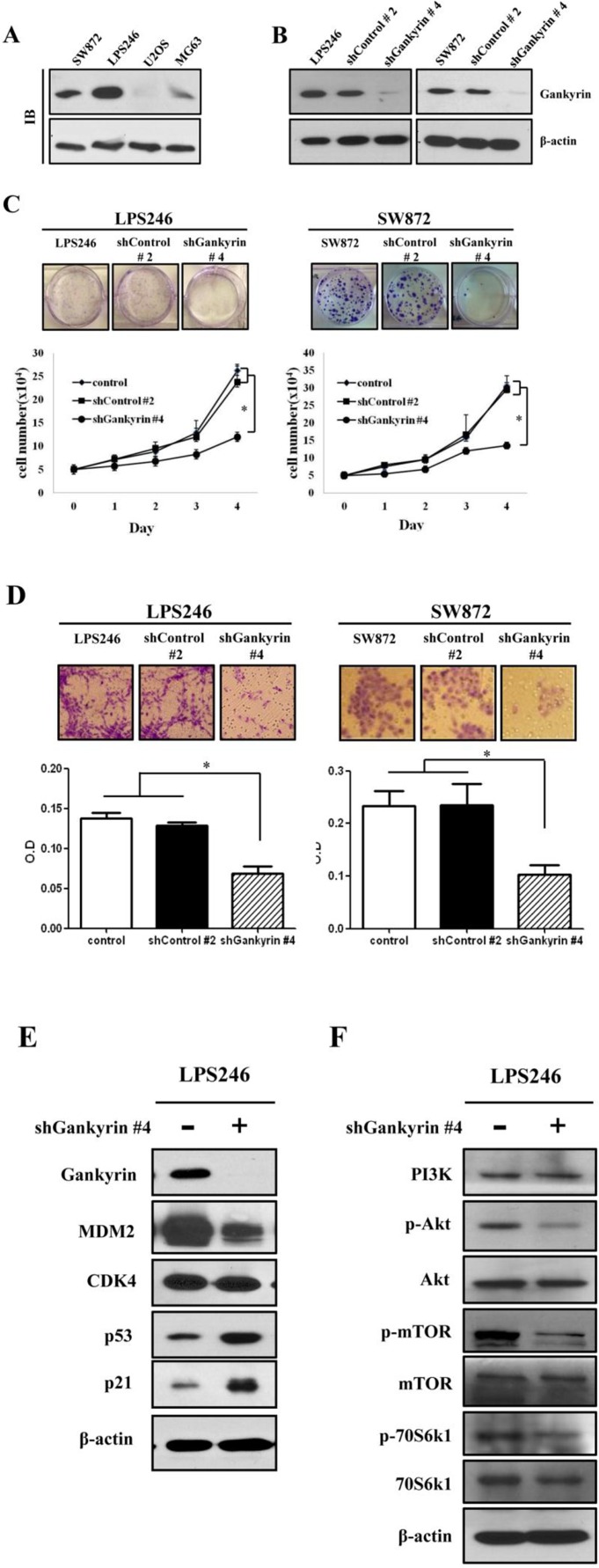
Effect of gankyrin inhibition on cell proliferation potentials (A) Gankyrin expression was detected by immunoblot in liposarcoma cell lines (LPS246 and SW872) and osteosarcoma cell lines (U2OS and MG-63). (B) Knockdown of gankyrin in DDLPS cell lines using the shGankrin lentiviral system (shGankyrin #4) was detected by immunoblot. shControl is non-target scramble shDNA (shControl #2). β-actin was used as an internal control. Colony formation and cell proliferation (C), migration (D) were detected in gankyrin knockdown DDLPS cell lines. Immunoblot assays in LPS246 cell line with gankyrin expression (E and F). Graphs represent the average of at least 3 repeated experiments ±SD. * denotes statistically significant effects (P < 0.05).

The molecular role of gankyrin has been previously reported in several types of cancers, especially HCC [15, 24, 25]. Because the biological role of gankyrin in DDLPS has not been previously reported, we evaluated gankyrin expression in the LPS246 and SW872 DDLPS cell lines. Consistent with the results obtained from DDLPS tissue, gankyrin was highly expressed in all DDLPS cell lines compared with the U2OS and MG63 osteosarcoma cell lines (Fig. 4A). We established endogenous gankyrin-silencing in each DDLPS cell line using the lentiviral shRNA system (Fig. 4B). The inhibitory role of gankyrin expression was confirmed at both the mRNA and protein levels, and its functional significance was also validated by colony forming unit (CFU) assays (Supplemental Fig. 1). Both LPS246 and SW872 DDLPS cell lines had significantly decreased cell proliferation, colony formation and migration ability following downregulation of gankyrin expression (Fig. 4 C and D). These results suggested that gankyrin may play an important role in promoting DDLPS cellular proliferation *in vitro*.

### Functional target of gankyrin in DDLPS cells

After evaluating the inhibitory effect of gankyrin expression in DDLPS cell lines, immunoblot assays were performed to identify the signaling modules that affect cell proliferation, colony formation and migration. The expression of MDM2 and CDK4 were tested after gankyrin inhibition. Although both proteins bind gankyrin, after gankyrin inhibition, the expression levels of MDM2 and gankyrin were markedly reduced, whereas the expression of CDK4 was not changed (Fig. 4E). Additionally, p53 and p21 were significantly increased by the inhibition of gankyrin expression and showed a pattern similar to that observed in DDLPS tissues (Fig. 2C). These data suggest that a major function of gankyrin may be to downregulate p53 via MDM2. Moreover, as might be anticipated by the inhibition of gankyrin expression, activated forms of AKT and mTOR (p-AKT and p-mTOR) were decreased. The phosphorylated form of p70S6k1, an mTOR downstream target involved with cell proliferation, was also diminished by shGankyrin expression in DDLPS cell lines (Fig. 4F). Taken together, these observations support the possibility that highly expressed gankyrin protein may participate in regulating the inhibition of p53 and AKT/m-TOR activation in DDLPS.

### Gankyrin inhibition reduces localized tumorigenesis of LPS cells *in vivo*

**Figure 5:**
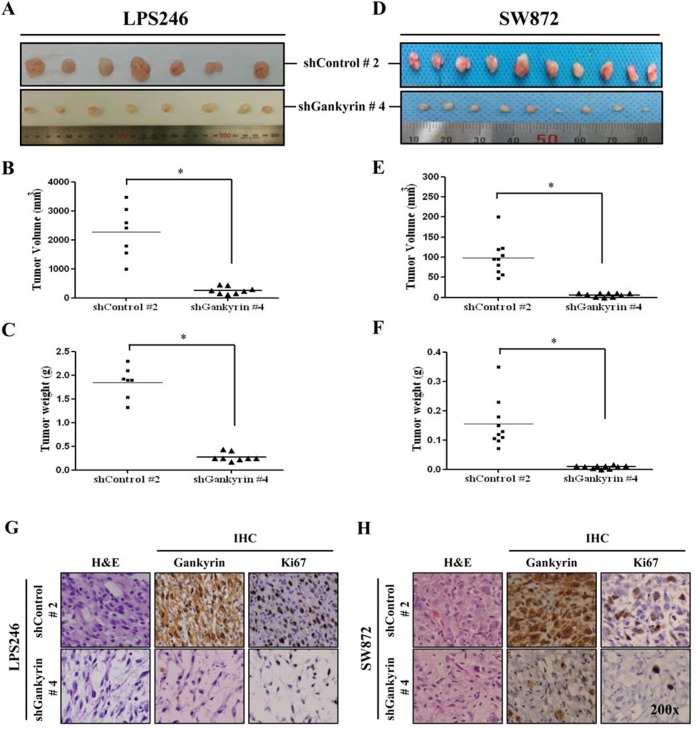
*In vivo* tumorigenesis of DDLPS cells along with gankyrin expression *In vivo* tumorigenesis of DDLPS cells, LPS246 (A) and SW872 (D) with or without gankyrin expression, were measured 6~8 weeks after the subcutaneous injection. The tumor volumes and weights of LPS246 cells (B and C) and SW872 cells (E and F) were detected, respectively. Representative image of H&E and IHC staining with gankyrin and Ki67 in shDNA-expressing LPS246 (G) and SW872 (H) xenograft tissues. * denotes statistically significant effects (P < 0.05).

To elucidate the role of gankyrin in DDLPS tumorigenesis *in vivo*, we established a local xenograft tumorigenesis model in immunodeficient NSG mice. The LPS246 and SW872 DDLPS cell lines with shControl and shGankyrin were used as tumorigenesis models. Although the tumorigenesis ability of DDLPS cell lines varied, *in vivo* tumorigenesis in the shControl (shControl #2) and shGankyrin groups (shGankyrin #4) showed significantly decreased tumor size (Fig. 5 A and D), volume (Fig. 5 B and E) and weight (Fig. 5 C and F). Furthermore, the results of the H&E and IHC staining demonstrated that gankyrin expression in xenograft tissues was properly visualized in the shControl and shGankyrin groups (Fig. 5 G and H). Ki-67 expression is indicative of tumor cell proliferation and revealed a positive correlation with gankyrin expression in each xenograft tissue sample. Taken together, these results suggest a functional significance for gankyrin expression in DDLPS tumorigenesis *in vivo*.

## DISCUSSION

LPS is the most abundant soft tissue sarcoma. Due to the low incidence of soft tissue sarcoma, only a few studies have identified robust target for optimal treatment strategies in this disease [10, 26-29]. Genetically, WD/DDLPS and pleomorphic LPS have varying degrees of chromosomal instability with heterogeneous and complex karyotypes [4, 30]. Most karyotypically complex liposarcomas have amplification of the 12q14 chromosome region that includes MDM2 and CDK4 gene [6, 7]. It was previously reported that MDM2 and CDK4 inhibition decreases the proliferation of LPS cell lines *in vitro* [31], and targeting these proteins are an ongoing clinical research strategy [32]. Moreover, because WDLPS and DDLPS respond modestly to systemic chemotherapy [33], the need to identify novel molecular targets for LPS therapy is critical. Several groups have performed tissue microarray and genomic analyses in an attempt to identify aberrantly regulated genes important to LPS generation. These genes include ZIC1, TOP2A, and AURKA, which have high expression levels across the liposarcoma spectrum [21-23]. Although DDLPS is more aggressive and has higher rates of metastasis and recurrence than WDLPS [34], both diseases have very similar cytogenetic features.

In this study, we examined the gankyrin expression level (Fig. 2) of 14 WD and/or DDLPS tissues (Table 1) after 2-DE and MALDI-TOF analysis. As shown in Figure 2, gankyrin is highly expressed in DDLPS and DDLPS sub-components at the mRNA and protein levels. However, the gankyrin expression levels in the WDLPS component of WD/DDLPS specimens (Fig. 2B) were slightly higher than those in WDLPS tissues (Fig. 2C, gankyrin IB). Accordingly, the WDLPS and DDLPS sub-components of WD/DDLPS specimens were usually physically separated after surgical resection when the LPS tissues were prepared for subsequent analysis (Fig. 1A). The imprecise identification of WDLPS and DDLPS sub-components during surgical excision may have contributed to some ambiguity in the separation of WDLPS and DDLPS. Moreover, other reports have demonstrated that the WDLPS sub-component of WD/DDLPS is molecularly similar to the DDLPS fraction of the tumor and that it may only be distinguished from pure WDLPS via CGH array, gene expression profiling, and similar molecular strategies [35, 36]. For this reason, the detection of additional protein expression levels related to gankyrin was performed using pure WDLPS and DDLPS tissues rather than WD/DDLPS specimens (Fig. 2C).

It is already known that p53 and Rb communicate via crosstalk pathways. Thus, we investigated the role of gankyrin and its possible relationship to these tumor suppressor molecules that impact cancer cell proliferation and survival. It is known that gankyrin expression is associated with the regulation of p53 and Rb in HCC [13]. However, it has also been reported that gankyrin positively regulates β-catenin signaling independent of p53 in HCC cell lines [37]. Moreover, in osteosarcoma cell lines, the knockdown of Rb enhances p53 destabilization in a gankyrin-dependent manner [12]. We observed that p53 and p21 were inversely correlated with gankyrin expression in WDLPS and DDLPS tissues. However, the expression of CDK4 as an LPS marker protein appears to be unrelated to gankyrin expression (Fig. 2C). Furthermore, Rb was not universally detected in our panel of WDLPS and DDLPS tissues (data not shown). This finding suggests that gankyrin expression corresponds strongly to p53 but not Rb expression in WDLPS and DDLPS tissues. Previous studies have shown that the downregulation of gankyrin induced apoptosis in HCC cells bearing wild-type p53 [12] and that p53 gene mutation is important in a variety of human cancers [38-40]. We also examined mutations of the p53 gene (Exon 2~11) in 14 LPS tissues using genomic DNA sequencing. Although the number of LPS tissues examined was not sufficient to reach definitive conclusions, no mutations of p53 were found in our 14 LPS tissues (data not shown).

Gankyrin expression levels have been positively associated with metastatic potential and are negatively associated with patient overall survival in hepatocellular carcinoma, esophageal squamous cell carcinoma, gastric cancer and colorectal cancer [19, 24, 25, 41]. However, to date, there have been no reports on the function of gankyrin in LPS. We verified that gankyrin overexpression correlated with tumor location, recurrence, metastasis (p=0.008, p=0.001 and p=0.009, respectively; Table 2) and overall survival (p<0.001; Fig. 3B) using immunohistochemical staining of gankyrin in a TMA of patient-derived WDLPS and DDLPS tissues (n=204; Fig. 3A). When we separately analyzed the overall survival of WDLPS and DDLPS based on gankyrin expression levels (Supplemental Fig. 2), gankyrin did not predict overall survival in the DDLPS samples (*p*-value = 0.782), whereas it predicted a slightly increased overall survival in WDLPS samples (*p*-value = 0.047). These results demonstrated that no significant prognosis in terms of overall survival in DDLPS in contrast with WDLPS. Additionally, these results suggest that a simple evaluation of gankyrin may not allow for an accurate prediction in the prognosis of LPS patients. However, although the prognostic significance of gankyrin is not easily verified in the DDLPS samples, these results demonstrated that gankyrin expression is useful indicator for the clinicopathological characteristics of WDLPS and DDLPS.

In HCC, the serine-threonine kinase Akt is considered a key factor in tumor survival and angiogenesis [42, 43]. Moreover, gankyrin may have a protective function in ER-stress-induced cell apoptosis via AKT phosphorylation and may contribute to HCC invasiveness and metastasis[18, 44]. Although aberrant Akt activation is a factor underlying leiomyosarcoma and WDLPS proliferation and tumorigenesis [45, 46], the role of gankyrin-mediated Akt signaling in LPS remains uncertain. We performed knockdown of gankyrin expression using a shDNA-expressing lentiviral system (Supplemental Fig. 1) based on previous reports in other cancers [24, 41, 44] to elucidate gankyrin functional affects in DDLPS cell lines. In the DDLPS cell lines LPS246 and SW872, gankyrin expression was greatly inhibited by shGankyrin viral infection (Fig. 4B). Using the LPS246 cells, we found that the levels of p-Akt, p-mTOR and p70S6K1 were reduced in gankyrin knockdown cell lines after confirmation of gankyrin, p53, p21, MDM2, and CDK4 expression. Moreover, gankyrin knockdown led to a reduction in cell proliferation, colony formation, and cell migration ability *in vitro* (Fig. 4 C and D) and reduced tumor formation *in vivo* (LPS 246: 017~03.45 g vs. 2.01~3.99 g, 120~455 mm^3^ vs. 2000~5115 mm^3^; SW872: 0.01~0.016 g vs. 0.12~0.35 g, 1~9.37 mm^3^ vs. 48~200 mm^3^; Fig. 5). These results strongly support the possibility that gankyrin may be an important oncogene in DDLPS.

In conclusion, we have demonstrated that gankyrin is dominantly expressed in DDLPS. Furthermore, high levels of gankyrin may positively correlate with the oncogenesis of liposarcoma and might be useful as a potential clinical predictive factor in WDLPS and DDLPS. Although further studies are needed to elucidate the mechanism(s) of gankyrin in LPS regulatory processes, these investigations suggest that gankyrin might also serve as a novel therapeutic target for WDLPS and DDLPS treatment.

## METHODS

### Liposarcoma tissue specimens

Fourteen patients who were diagnosed with WDLPS, DDLPS and WD/DDLPS and treated at Samsung Medical Center between September 2009 and May 2011 were evaluated for this study. Informed consent was obtained from all patients. The project was approved by the Samsung Medical Center institutional review board (Seoul, Korea). LPS and normal adipose tissues were obtained at the time of surgical resection. LPS tissues were used after confirmation of MDM2 expression and stored at −80 °C until analysis. The clinical characteristics of the study patients are summarized in supplemental table 1.

### Tissue microarray (TMA)

TMAs containing 204 human WDLPS and DDLPS tissues were prepared. TMA construction and clinical information were approved by the University of Texas MD Anderson Cancer Center (UTMDACC) IRB. Tissue sections 5 μm thick were cut from TMA blocks and used for immunohistochemical staining. The quantitative analysis of LPS samples was conducted by assigning a score depending on the overall gankyrin staining intensity (0: no staining, 1: low intensity, 2: moderate intensity, 3: high intensity). The staining scores were assigned by a soft tissue pathologist (Alexander J Lazar).

### Histological and immunohistochemical (IHC) staining

Formalin-fixed and paraffin-embedded tissues were stained with hematoxylin and eosin for histological observation and analyzed by IHC staining. Briefly, heat-induced antigen retrieval was performed using 0.01 M sodium citrate buffer in a microwave for 15 minutes. After quenching of endogenous peroxidase and blocking in 3 % goat serum, the tissues were incubated with primary polyclonal anti-gankyrin antibody (Santa Cruz Biotechnology), anti-Mdm2, anti-CDK4 (Invitrogen) and anti-Ki-67 (Dako). After three TBST washes followed by secondary antibody, peroxidase activity was detected with 32-diaminobenzidine-tetrahydrochloride (DAB, Dako). Negative and positive controls were used to establish the specificity of these reactions.

### 2-Dimensional gel electrophoresis and image analysis

LPS tissues were resuspended in lysis buffer (8 M urea, CHAPS, 0.5 % IPG buffer pH 3-10), and disrupted using an ultrasonicator before centrifugation. The protein separation was performed in duplicate for each sample. For each sample, 1 mg of protein was loaded on an IPG strip (18 cm, pH 3–10 nonlinear) that was rehydrated overnight. Isoelectric focusing (IEF) was performed using the following parameters with the Multiphor II system (Amersham Biosciences): 500 V for 1 h, 1000 V for 1 h, 5000 V for 1 h, 8000 V (gradient) for 1 h, and finally, 8000 V for a total of 45 kV h. After IEF, the IPG strips were immediately equilibrated in 10 ml equilibration solutions (50 mM Tris–HCl, pH 8.8, 6 M urea, 30 % glycerol, 2 % SDS, 0.002 % bromophenol blue) with gentle shaking for 15 min. Vertical SDS-PAGE was run with laboratory-made homogenous 10 % acrylamide gels in an Ettan DALTII apparatus (Amersham Biosciences). The gels were stained with coomassie blue, and image analysis was performed using the Image Master 2-D Platinum software (Amersham Biosciences) according to the protocols provided by the manufacturer.

### Matrix-assisted laser desorption/ionization-time of flight mass spectrometry/mass spectrometry

The protein spots of interest were excised manually from the preparative gels, destained in 50 mM NH_4_HCO_3_/acetonitrile (50:50) and dried by vacuum centrifugation. To rehydrate the tissues, the gel pieces were incubated in 100 mM ammonium bicarbonate and 10 mM DTT for 1 h at 56°C. Alkylation was performed in 100 mM ammonium bicarbonate and 55 mM iodoacetamide for 40 min at room temperature in the dark. After dehydration in acetonitrile, the gel pieces were dried and digested with sequencing grade trypsin (enzyme:substrate (w/w) ratio > 1:20) (Promega) at 37°C overnight in 50 mM ammonium bicarbonate. After a repeated process of hydration–dehydration and sonication, the resulting tryptic peptides were dissolved in 0.5 % trifluoroacetic acid (TFA) solution. The peptides were then desalted using a ZipTipC18 (Millipore) tip. The peptides were eluted directly onto the MALDI target with an α-cyano-4-hydroxy-cinnamic acid (CHCA) matrix solution (10 mg/ml CHCA in 0.5 % TFA/50 % acetonitrile (1:1, v/v). All mass spectra were acquired in reflection mode by a 4700 Proteomics Analyzer (Applied Biosystems).

### Cell culture

HEK293T (human embryonic kidney 293T) cells, SW872 (DDLPS cells), U2OS and MG63 (osteosarcoma cells) were purchased from the American Type Culture Collection. Human DDLPS cell lines LPS246 and primary LPS cells were isolated and cultured as previously described. The cells were then used for FISH analysis [23]. The cells were maintained in DMEM-high glucose media with 10 % fetal bovine serum (FBS), 100 units/ ml penicillin plus 100 μg/ml streptomycin (1X P/S) and incubated at 37 °C in 5 % CO2.

### MDM2 FISH analysis

FISH was performed with cells isolated from WD/DDLPS tissue using a MDM2 probe (Kreatech) that was specific for the centromere region of chromosome 12q15. Briefly, 10 μl of probe or probe-mix per 22 × 22 mm was applied to the field to fix the cells. The cells were covered by a glass cover slip and sealed with Fixogum or Rubber Center. The sample was denatured and probed on a hot plate at 75°C for 5~10 min. The cells were incubated overnight at 37°C in a humidified chamber. The slides were washed in 1x wash buffer (2x SSC/0.1 % Igepal) at room temperature and dehydrated in 70 %, 85 % and 100 % ethanol for 1 min each. After air drying at room temperature, 15 μl of DAPI counterstain (LK-095A) was applied and the preparations were covered with a glass cover slip. Microscopy was performed, and 200 nuclei per slide were counted.

### Gene knockdown by shRNA lentivirus

Lentivirus plasmid vectors pLKO.1-puro and pLKO.1-puro containing MISSION shRNA targeting gankyrin (shGankyrin) or non-targeting shRNAs (shControl) were purchased from Sigma-Aldrich. Five distinct sequences per gene were assessed for knockdown (clone #0: TRCN0000290090, #1: TRCN0000290021, #4: TRCN0000058074, #5: TRCN0000058075, #7: TRCN0000058077). The shControl sequence is not known to target any human genes (SHC002) and served as a negative control. The infectious viral supernatants were collected in viral harvest medium 24 hours after transfection. To knockdown gene expression, the cells were infected with lentivirus using 8 μg/ml polybrene (Sigma-Aldrich) to increase infection efficiency. The infected cells were selected with 1.5~3.0 μg/ml puromycin (Sigma-Aldrich). Gankyrin knockdown was measured by RT-PCR and immunoblot assays.

### Colony formation and cell proliferation assay

To examine colony formation, 500 cells per well were plated into 6-well plates and grown in DMEM-high glucose medium for 10 days. The cells were then stained with 0.5 % crystal violet solution for 30 min at room temperature. Pictures were digitally captured, and the number of colonies was counted. Cell proliferation was counted every day after seeding cells (5×10^4^) for 4 days.

### Migration assay

Polycarbonate filters with 8-μm pores (Becton Dickinson Labware) in 24-well tissue culture plates were used for modified Boyden chamber migration assays. The lower chamber compartments contained DMEM supplemented with 1 % fetal bovine serum as a chemoattractant. The cells (5×10^4^) were seeded in the upper well and incubated at 37 °C. After incubation, filters were fixed with acetic acid and stained with 0.2 % crystal violet. The absorbance was measured at 650 nm with a microplate reader (Molecular Devices Corporation).

### RNA preparation and PCR

Total RNA was extracted from LPS tissues with Trizol reagent (Invitrogen). cDNA was synthesized with superscript III and oligo(dT) (Invitrogen) according to the manufacturer's instructions. PCR products were normalized using β-actin amplification. The primer sequences and expected sizes are shown below. The human gankyrin forward primer was 5′-GCAACTTGGAGTGCCAGTGAA - 3′, and the reverse primer was 5′- TCACTTGAGCACCTTTTCCCA - 3′, and generated a product of 198 bp. The human β-actin forward primer was 5′-CTACGTCGCCCTGGACTTCGAGC - 3′, and the reverse primer was 5′-GATGGAGCCGCCGATCCACACGG - 3′, and generated a 420 bp product. PCR amplifications were performed as follows: 30 cycles of 60 s at 94 °C, 60 s at 55 °C and 60 s at 72 °C. The PCR products were analyzed in 1 % agarose gel.

### Immunoblot assay

Immunoblot assays were performed using standard methods. Briefly, total protein (10~25 μg) from LPS tissues and cells was separated by SDS-PAGE and transferred onto PVDF membranes. The membranes were blocked with 5 % nonfat dried milk or 5 % BSA and then blotted with relevant primary antibodies. HRP-conjugated secondary antibodies were detected using an enhanced chemiluminescence (ECL) kit (Ab Frontier). The antibodies used in these studies are as follows: Gankyrin, p53, p21 (Santa Cruz Biotechnology), Mdm2 (Invitrogen), CDK4 (Invitrogen), PI3K, AKT, phospho-AKT, m-TOR, phospho-m-TOR, p70S6 Kinase, phospho-p70S6 Kinase (Cell signaling), and β-actin (Sigma).

### Establishment of an *in vivo* tumorigenesis mouse model

LPS246 (2×10^6^) and SW872 (1×10^7^) cells were subcutaneously injected into 6-week-old NSG mice (each n=10). The mice were sacrificed 6~8 weeks after implantation. Xenograft explants of tumor tissues were confirmed by histological and immunohistochemical staining.

### Statistical Analysis

The chi-squared test or the Fisher's exact test was used to compare categorical variables. The survival rates were calculated using the Kaplan-Meier method, and the survival curves were compared using log-rank tests. Statistical analyses were performed with PASW Statistics 18 software (IBM Corporation, Armonk, NY).

## SUPPLEMENTARY MATERIAL, FIGURES AND TABLE


