# Supplementary material for: Gankyrin is a predictive and oncogenic factor in well-differentiated and dedifferentiated liposarcoma
Source: Oncotarget. 2014 Aug 21;5(19):9065–78. doi: 10.18632/oncotarget.2375 (PMC4253419; doi:10.18632/oncotarget.2375)
Supplement: Supplementary file 1 [file oncotarget-05-9065-s001.pdf]

Gankyrin is a predictive and oncogenic factor in well-differentiated and dedifferentiated liposarcoma

Supplementary Material

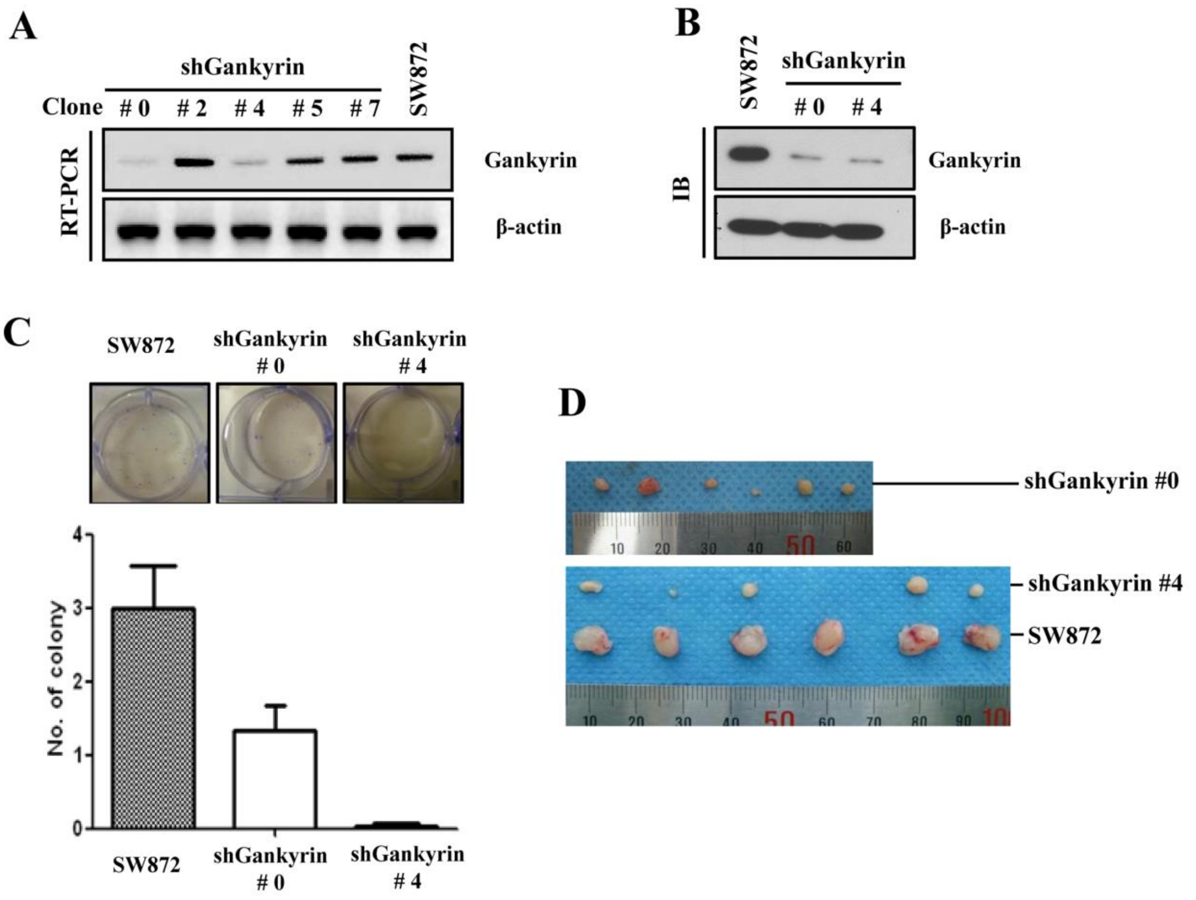

Supplemental Figure 1: Screening of shDNA clone for the gankyrin knockdown using a lentiviral system. Representative figure for the RT-PCR and immunoblot assay of gankyrin. SW-872 cells were transduced with five types of lentiviruses containing shGankyrin clones (A), and two types of shGankyrin clones (shGankyrin #0 and #4) were selected for the knockdown of gankyrin (B). (C) Colony forming assay (CFU) of SW872 with or without two shGankyrin clones. (D) *In vivo* tumorigenesis of SW872 cells and SW872 shGankyrin clones. Tumor mass was measured 6~8 weeks after the subcutaneous injection.

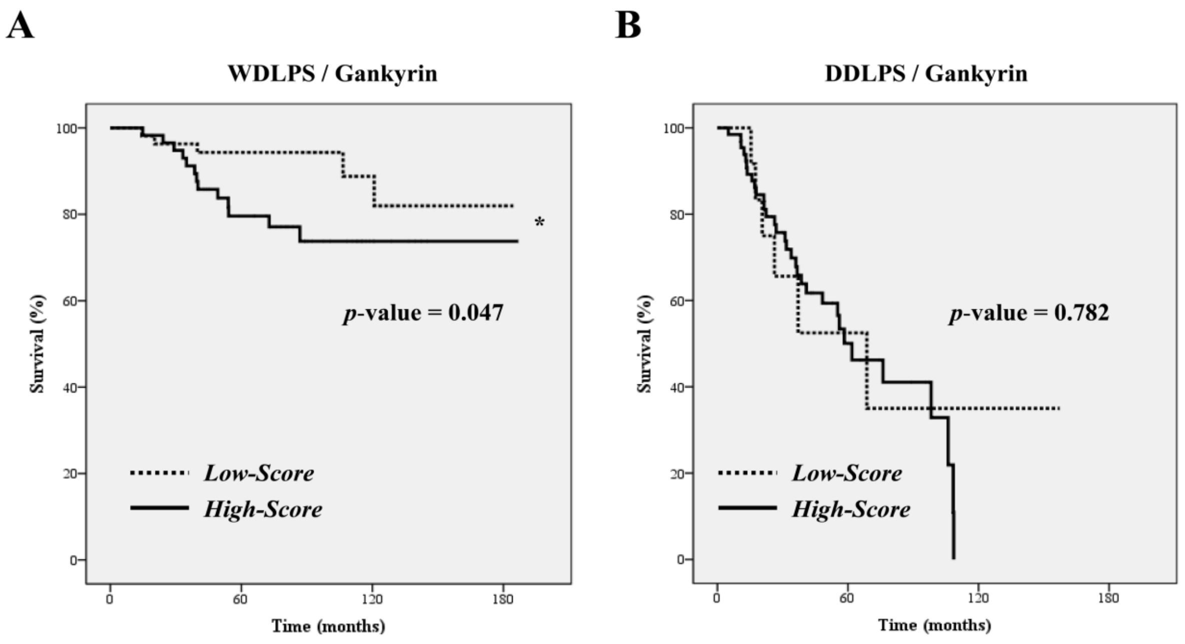

Supplemental Figure 2: The analysis of overall survival in 204 LPS samples stratified according to WDLPS and DDLPS and gankyrin expression scores. Each WDLPS (A) and DDLPS (B) sample was separately analyzed for overall survival and gankyrin expression score. * denotes statistically significant effects ($p < 0.05$).

Supplemental Table 1

Clinical characteristics of liposarcoma patients

NO.	List	Age (y)	Sex	Subtype	Primary / Reccurent	Site	Size (cm)	Necrosis	MDM2 (IHC)
1	LPS 131	67	M	WD/DDLPS	Primary	Retroperitoneum	10x6	absent	Positive
2	LPS 851	58	F	WD/DDLPS	Primary	Kidney, small bowel	10x9.5x8	>50%	Positive
3	LPS 719	63	M	WD/DDLPS	Primary	Retroperitoneum	12x9x7.5	<50%	Positive
4	LPS 850	40	F	WD/DDLPS	Primary	Retroperitoneum	10x10	absent	Positive
5	LPS 391	69	M	WD/DDLPS	Primary	Retroperitoneum	12x7	absent	Positive
6	LPS 645	58	M	WD/DDLPS	Reccurent	colon and small bowel	16x11x10	absent	Positive
7	LPS 163	44	M	WDLPS	Reccurent	Omental	28x23	present	Positive
8	LPS 249	45	M	WDLPS	Reccurent	Retroperitoneum and omentum	7.6x6.5	absent	Positive
9	LPS 331	44	F	WDLPS	Primary	Retroperitoneum	36x24x7	absent	Positive
10	LPS 809	63	M	WDLPS	Reccurent	inguinal	7x5x4	absent	Positive
11	LPS 822	52	M	DDLPS	Reccurent	Retroperitoneum	10x8x7	<50%	Positive
12	LPS 706	50	F	DDLPS	Reccurent	Retroperitoneum, small bowel	49x30x11	<50%	Positive
13	LPS 697	38	M	DDLPS	Primary	Retroperitoneum	15x12x9	absent	Positive
14	LPS 216	68	M	DDLPS	Reccurent	Kidney	14x13x13	present	Positive
